# Sudden Cardiac Arrest in Athletes: A Primary Level of Prevention

**DOI:** 10.7759/cureus.30517

**Published:** 2022-10-20

**Authors:** Keerthana Prakash, Kiran Maee Swarnakari, Meena Bai, Mohana Priya Manoharan, Rabab Raja, Aneeque Jamil, Denise Csendes, Sai Dheeraj Gutlapalli, Aditya Desai, Darshi M Desai, Pousette Hamid

**Affiliations:** 1 Internal Medicine, California Institute of Behavioral Neurosciences & Psychology, Fairfield, USA; 2 Internal Medicine, University of California Riverside School of Medicine, Riverside, USA; 3 Neurology, California Institute of Behavioral Neurosciences & Psychology, Fairfield, USA

**Keywords:** sports, screening, prevention, athletes, sudden cardiac arrest

## Abstract

Primary prevention of sudden cardiac arrest (SCA) refers to the use of pharmacological or interventional therapy and healthy lifestyle modifications to prevent sudden cardiac death (SCD) in patients who have not experienced symptomatic, life-threatening persistent ventricular tachycardia or ventricular fibrillation or SCA but are considered to be at a higher risk. This review provides an overview of the physiological heart changes and distinct electrical manifestations, the etiology of SCA, and screening methods and interventions for the prevention of SCA in athletes. The American College of Cardiology and the American Heart Association (AHA) Guidelines recommend screening with a 14-point history and physical examination. In most cases, a thorough clinical evaluation along with an ECG is sufficient for screening. Athletes with heart diseases leading to SCD are urged not to compete. Further decisions are taken following the European Society of Cardiology and the AHA's current workout recommendations. Early detection of cardiac disease allows for individualized risk evaluation and treatment, which has been shown to reduce mortality rates in athletes.

## Introduction and background

Regular aerobic physical activity improves exercise capacity and helps prevent a range of chronic diseases, including cardiovascular disease (CVD), diabetes, cancer, and osteoporosis, on both primary and secondary levels. Surprisingly, athletes with dormant electrical, structural, and acquired cardiovascular abnormalities are more vulnerable to exercise-related sudden cardiac death (SCD) [1]. An SCD is defined as *a nontraumatic, unexpected death occurring due to a cardiovascular cause within one hour of the onset of symptoms in a healthy subject*. An athlete is a person who engages in regular physical training and competes in official sports to achieve excellence and success. The desire to achieve or exceed a performance limit is linked to an athlete's ability to put up maximal effort during strenuous sports activity. As a result, the cardiovascular system is put under extreme stress throughout training and competition.

In competitive athletes, the incidence of life-threatening cardiovascular events, including SCD, is high, with rates ranging from 0.76 per 100,000 to 13 per 100,000 [2-5]. SCD is 2.8 times more common in adolescents and young adults who participate in athletics than in those who do not [6]. In athletes, the risk of SCD increases with age and is higher in males [7]. Sudden cardiac arrest (SCA) is the most common medical cause of sudden death in athletes at any age. However, in athletes over 35 years of age, coronary artery disease (CAD) is the most common cause of SCD, whereas genetic diseases are a major cause of SCD in young athletes [6,8,9].

SCD in sports is a devastating occurrence that affects friends, family, communities, and athletic organizations [10]. Everyone instantly wonders what could have been done specifically to avoid death. In sports cardiology, preventing SCD in athletes is a key constitutional duty [11]. SCD can be caused by a variety of structural, electrical, and acquired CVD, many of which can be detected early in life and treated with lifestyle interventions, pharmacotherapy, and device therapy [1]. The goal of this review is to concentrate on CVD screening and interventions for preventing SCD in sports.

Athletic field arrhythmic cardiac arrest is caused by a combination of physical exercise and underlying CVD, rather than by exercise alone. This finding sets up the idea that physicians and sports trainers should check athletes regularly to identify those with potentially fatal cardiac diseases and protect them from the higher risk of SCD [6]. The purpose of a pre-participation physical examination (PPE) is to ensure that athletes are healthy and safe during competition by screening them for injuries and illnesses [12].

## Review

Physiological changes in an athlete's heart

Hemodynamic, electrophysiologic, and structural alterations occur in normal cardiac tissue as a result of consistent, regular exercise [13,14]. The oxygen consumption of muscle tissue rises significantly during vigorous aerobic activity, requiring an increase in cardiac output to supply the demands. Aerobic exercise increases the left ventricular mass, increases the ventricular stroke volume, and increases the cardiac output [15] and the heart rate during exercise with time. Reduced resting heart rate is due to increased vagal tone [16-18].

In well-trained athletes, physiologic hypertrophy of the heart in response to cardiovascular conditioning is common [19]. It was shown to be predominantly eccentric in endurance athletes (dynamic/aerobic) and more frequently concentric in strength athletes (isometric/anaerobic). Athletes have a 15%-20% thicker left ventricular wall thickness (LVWT) and a 10%-15% larger left ventricle size compared to the general population. These are adaptive systems that can regress if they are detrained. LVWT > 13 mm and left ventricle diameter > 60 mm are uncommon in healthy athletes [20,21].

The second structural adaptation in trained athletes, primarily in endurance sports, is left atrial dilatation. Associated left ventricle cavity enlargement and volume overload can explain left atrial enlargement (cutoff values: 46 mm in females and 50 mm in males) [22], which explains the higher incidence of supraventricular arrhythmias seen in adult athletes [23].

There are minor atrioventricular pressure gradients at rest when heart flow is minimal. When the atrioventricular valves are closed during systole, the high-flow state causes significant atrial filling and a rise in pressure. Through the pulmonary circulation, this high left atrial pressure builds up, causing the right ventricle afterload elevation. Right ventricular dilation is determined by the increased right ventricle afterload [24].

Sudden cardiac arrest in athletes: Etiology 

The most frequent factors contributing to SCD in young and master athletes are mentioned in Tables 1, 2.

**Table 1:** Causes of SCA in young athletes (age < 35 years). ARVD/C, arrhythmogenic right ventricular dysplasia/cardiomyopathy; BrS, Brugada syndrome; CPVT, catecholaminergic polymorphic ventricular tachycardia; DCM, dilated cardiomyopathy; HCM, hypertrophic cardiomyopathy; LQTS, long QT syndrome; SCA, sudden cardiac arrest

Inherited causes
1. Primary inherited arrhythmia syndromes [25]: LQTS, BrS, CPVT
2. Cardiomyopathies: HCM [25], DCM [25], ARVD/C [5]
3. Congenital coronary artery anomalies [26]
4. Idiopathic concentric left ventricular hypertrophy [27]
5. Marfan’s syndrome with aortic rupture [27]
Acquired causes
1. Infection (myocarditis) [27]
2. Trauma (commotio cordis) [28]
3. Toxicity (illicit/performance-enhancing drugs) [29]
4. Mitral valve prolapse [27]

**Table 2:** Causes of SCA in master athletes (age > 35 years). ARVD/C, arrhythmogenic right ventricular dysplasia/cardiomyopathy; HCM, hypertrophic cardiomyopathy; SCA, sudden cardiac arrest

Causes of SCA in master athletes (age > 35 years)
1. Atherosclerotic coronary artery disease (80%) [30]
2. Idiopathic left ventricular hypertrophy
3. Heritable cardiomyopathies [13]: HCM and ARVC
4. Primary electrical disease with no specific cause identified [13]
5. Myocarditis [13]

SCD appears to be unaffected by the athletic competition level (high school students, college students, or professionals) [31]. Cardiovascular maladaptation such as atrial fibrillation, coronary artery calcification, and myocardial fibrosis has been linked to high-volume, high-intensity exercise training [32]. Overtraining, inadequate regeneration, substance misuse, and underlying familial, genetic, subsequent, and other variables, all contribute to the occurrence of SCD [33].

The underlying arrhythmogenic substrate interacts adversely with the catecholamine surge during exercise. Dehydration, hyperpyrexia, electrolyte imbalance, and enhanced platelet aggregation can all occur as a result of intense exercise, facilitating ventricular tachycardia or fibrillation even more [1].

In patients with structural or electrical abnormalities, exercise may act as a trigger for life-threatening arrhythmias [25]. Patients with hypertrophic cardiomyopathy will have a relatively benign course with minimal symptoms, although sports can cause cardiac arrest [34]. Depolarization abnormalities, early CAD, congenital coronary malformations, and Marfan syndrome are all potential hidden CVDs that can lead to life-threatening ventricular arrhythmias (VAs) during exercise [35]. Commotio cordis is a concussion of the heart caused by nonpenetrating physical trauma to the anterior chest, which is an uneventful outcome. Due to myocardial damage or the mechanoelectrical activation of a ventricular tachyarrhythmia, commotio cordis can occasionally lead to fatal cardiac arrest [28].

Abuse of doping and performance-enhancing drugs has been associated with CVD. Excessive hypertrophy has been linked to substances such as anabolic-androgenic steroids (AASs), growth hormone, thyroid hormone, coffee, and cocaine. In addition to its performance-enhancing impact, excessive usage can lead to adverse effects such as arrhythmia and hypertension [36]. Ergogenic aids are widely used and abused to enhance performance, body weight, aggression, mental attention, and physical strength while delaying exhaustion and pain desensitization [37]. AASs are synthetic testosterone derivatives that athletes may abuse to increase muscle mass and athletic performance and improve physical appearance [38]. AAS misuse is not limited to competitive athletes; it has been documented at all levels, from elementary school children to professional athletes, and is particularly common among bodybuilders and resistance athletes. Androgens have been shown to have a significant impact on lipoprotein metabolism, resulting in a more atherogenic profile that increases the risk of CAD, myocardial infarction, and SCD [39]. Ephedrine is another potent sympathomimetic drug that can result in cardiomyopathy. AAS can also cause hypertension, platelet hyperactivity, and vasoreactivity consequences. Blood clotting and fibrinolysis are adversely impacted [37].

CAD is more common in middle-aged athletes than in inactive people [40], with the most active athletes having the highest plaque prevalence [41]. Athletes who have CAD have a higher risk of death and acute cardiac events than athletes who do not have CAD. The disruption of massive plaques caused by excessive physical exercise, followed by ischemia, is the mechanism of SCD in athletes with CAD.

Primary preventive measures

Pre-participation screening (PPS) is a systematic method of evaluating athletes before competition or participation to detect any existing cardiac pathology that could put an athlete at risk of SCD, as well as any other significant health concerns. The concept of screening is that athletes at high risk of sudden death may be predicted and that an intervention, usually involving a limitation on competitive or high-intensity sports, will significantly lower the chance of mortality in these individuals. Screening must be sensitive and allow for the detection of the most common causes of sudden death for *prediction* to be effective. False-positive results can lead to unnecessary diagnostic testing and, unfortunately, the restriction of athletes who are at low risk [13].

A screening program should focus on common, treatable medical conditions and use a suitable, cost-effective test that can accurately detect the condition, and this can lead to an intervention that can reduce complications of the condition without causing adverse effects by not exposing those who will not benefit from the intervention [42].

All routine screening programs should include a medical and family history, as well as a physical examination [11,43].

Medical History

Good history-taking and clinical judgment remain important not only for risk categorization purposes but also because symptoms may be the only evidence sometimes. The following history should be thoroughly assessed: 

The symptoms that are most strongly suggestive of arrhythmias are recurrent syncope, palpitations, lightheadedness, dyspnea, and chest pain. The symptoms of underlying heart diseases such as coronary, valvular, and congenital heart diseases; cardiomyopathy; channelopathies; conduction disorders; and pacemakers. Other exogenous triggers include a history of doping and performance-enhancing drug abuse, smoking, fever, alcohol consumption, and hypertensive crisis [44].

Family History

To achieve a definitive diagnosis of an electrical or structural heart illness, such as channelopathies and cardiomyopathies, a family history of hereditary cardiac disorders or SCD is an important step in the integrated flowchart [45].

Athletes may not report or underreport symptoms to make the screening procedure more difficult because they are concerned about losing their training, sponsorship, or other professional opportunities [46].

Physical Examination

Along with a standard cardiac examination, it's vital to rule out congenital lesions that can be detected by specific heart murmurs or pulse and BP differences in the hands and feet (e.g., coarctation).

Many syndromes are associated with CVD (e.g., Williams syndrome, connective tissue disease symptoms, Turner syndrome, and Marfan syndrome). This is relevant when assessing Paralympic athletes [47].

For pediatric sports, nutritional evaluation is important. Furthermore, adrenergic overstimulation, which is common with the use of energy drinks, might make nutritional supplements arrhythmogenic [48].

ECG:* *An ECG is a much more effective screening tool than cardiac history and auscultation/inspection in detecting cardiovascular abnormalities, also emphasizing that further tests are required before approval for participation in sports can be given [37].

Even among expert ECG interpreters, interobserver variability remains significant, implying that interpreting ECGs requires considerable knowledge [49]. When interpreting an ECG, look for heart rate, time intervals, the heart axis, negative precordial T waves, and evidence of hypertrophy. Regular and long-term vigorous exercise (at least four hours per week) is linked to physiological heart changes and distinct electrical manifestations. In athletes, these ECG readings are considered normal (Table 3) [50]. And abnormal ECG findings are depicted in Table 4 [50].

**Table 3:** Normal and borderline ECG findings in athletes. Athletes with normal ECG findings who are asymptomatic and have no family history of hereditary cardiac diseases or SCD do not need any further evaluation. If two or more borderline findings are present, then further evaluation is necessary to look into any pathologic cardiovascular diseases related to SCD in athletes. AV, atrioventricular; RBBB, right bundle branch block; SCD, sudden cardiac death

Normal ECG findings
Sinus bradycardia or arrhythmia
Ectopic atrial or junctional rhythm
T-wave inversion V1-V3 (age < 16 years)
Mobitz type 1, secondary AV block
Increased QRS voltage for ventricular hypertrophy
Incomplete RBBB
Early repolarization/ST segment elevation
ST elevation followed by T-wave inversion V1-V4 in athletes
Primary AV block
Borderline ECG findings
Right axis enlargement
Right atrial deviation
Left axis deviation
Left axis enlargement
Complete RBBB

**Table 4:** Abnormal ECG findings in athletes. ARVC, arrhythmogenic right ventricular cardiomyopathy; CAD, coronary artery disease; CMR, cardiovascular magnetic resonance; DCM, dilated cardiomyopathy; HCM, hypertrophic cardiomyopathy; LVNC, left ventricular noncompaction; MI, myocardial infarction; SAECG, signal-averaged ECG; WPW, Wolff Parkinson White syndrome; LBBB, left bundle branch block; LQTS, long QT syndrome; QTc, corrected QT interval

ECG abnormality	Probable disease	Recommended evaluation
Complete LBBB	DCM, HCM, LVNC, sarcoidosis, and myocarditis	Echocardiography and CMR (with stress perfusion study)
ST-segment depression	HCM, DCM, LVNC, ARVC, and myocarditis	Echocardiography
T-wave inversion in the anterior leads	DCM and ARVC	Echocardiography, CMR, exercise the ECG test, minimum 24-hour ECG monitor, and SAECG
T-wave inversion isolated to the inferior leads	Myocarditis, LVNC, HCM, and DCM	Echocardiography
T-wave inversion in the lateral or inferolateral leads	ARVC (with predominant LV involvement), Myocarditis, HCM, DCM, and LVNC	Echocardiography, CMR, exercise the ECG test, and minimum 24-hour ECG monitor
Epsilon wave	ARVC	Echocardiography, CMR, exercise the ECG test, minimum 24-hour ECG monitor, and SAECG
Ventricular preexcitation	WPW syndrome	Exercise the ECG test and echocardiography
Pathologic Q waves	LVNC, DCM, HCM, prior MI, and myocarditis	Echocardiography, CAD risk factor assessment, repeat ECG for septal (V1-V2) QS pattern; aforementioned investigations recommended if septal Q waves are persistent
Profound nonspecific intraventricular conduction delay >=140 ms	HCM, DCM, LVNC, and ARVC	Echocardiography
Multiple premature ventricular contractions	LVNC, ARVC, HCM, DCM, sarcoidosis, and myocarditis	Echocardiography, 24-hour ECG monitor, and exercise the ECG test
Two or more borderline ECG	Myocardial disease	Echocardiography
Advanced second- or third-degree atrioventricular block	Myocardial or electrical disease	Echocardiography, minimum 24-hour ECG monitor, and exercise the ECG test
Profound sinus bradycardia <30 beats/minute		Repeat ECG after mild aerobic activity
Prolonged QTc	LQTS	Repeat resting ECG on a separate day, review QT-prolonging medication, and acquire ECG of first-degree relatives if possible
Brugada type 1 pattern	Brugada syndrome	Referral to a cardiologist or a heart rhythm specialist
Profound 1 atrioventricular block >=400 ms	Myocardial or electrical disease	Repeat ECG after mild aerobic activity and exercise the ECG test
Ventricular arrhythmias	Myocardial or electrical disease	Exercise the ECG test, echocardiography, minimum 24-hour ECG monitoring
Atrial tachyarrhythmias	Myocardial or electrical disease	Exercise the ECG test, echocardiography, and minimum 24-hour ECG monitoring

Exercise Testing

An exercise ECG test is useful for determining the BP response to exercise, the presence of exercise-induced arrhythmias, and the functional capacity required for exercise training. Exercise testing may also be important in predicting the functional capacity of the heart and lungs in the elderly who have recently engaged in moderate or severe exercise. It enables the evaluation of premature ventricular beats (PVBs) behavior as workload increases, as well as other aberrant results that may indicate an underlying heart illness, such as ST segment alterations. To maximize the test sensitivity of unmasking adrenergic-dependent PVBs or myocardial ischemia occurring at high workloads, stress testing is recommended for athletes with symptoms concerning ischemic heart disease. Athletes should be tested for endurance rather than stopping at 85% of their theoretical maximal heart rate [51].

Echocardiography:* *The standard screening method for determining coronary artery takeoff from the aorta can detect congenital coronary artery anomalies, which are the most common cause of ischemia-induced VAs and SCD in athletes [52]. Echocardiography is also used to examine systolic function, diastolic filling pattern, ventricular wall thickness, chamber size, and wall motion anomalies that could indicate ischemic heart disease, valvular abnormalities, congenital heart disorders, and cardiomyopathies [51].

Cardiac magnetic resonance (CMR): It has the unique ability to identify and quantify myocardial tissue abnormalities such as edema, fatty infiltration, or replacing-type fibrosis using the late gadolinium enhancement technique. It also allows accurate evaluation of cavity dimensions, wall thickness, global systolic function, and regional wall motion abnormalities of both ventricles. CMR has become a critical test for the evaluation of athletes with PVBs with high-risk morphological abnormalities on resting 12-lead ECG or exercise testing, as it has been shown that contrast-enhanced CMR can detect the presence of a nonischemic left a ventricular scar, which is otherwise missed by echocardiography. When echocardiography is unclear, CMR can be helpful, especially if the ectopic QRS is long (>130 ms) and has a right bundle branch block/superior axis pattern, indicating that it originated from the inferolateral left ventricular wall [51].

Genetic testing: To diagnose a heritable etiology that may be genetically transmissible, genetic testing can be used in relatives of people with autopsy-negative SCD or unexplained cardiac arrest [53,54]. This is a useful strategy for identifying asymptomatic relatives who have a genotype-positive test (Table 5). It was first suggested that molecular autopsy may be used to ascertain the cause of death in SCD patients. This method can detect mutations in genes linked to cardiomyopathy and channelopathy [55], and genetic counseling can help.

**Table 5:** Molecular genetic testing of mutations in genes. AD, autosomal dominant; AR, autosomal recessive; BrS, Brugada syndrome; CPVT, catecholaminergic polymorphic ventricular tachycardia; *CASQ2*, calsequestrin 2; DCM, dilated cardiomyopathy; hERG, The human Ether-à-go-go-Related Gene; HCM, hypertrophic cardiomyopathy; *KCNQ1*, potassium voltage-gated channel subfamily Q member 1; *KCNH2*, potassium voltage-gated channel subfamily H member 2; *KCNE5*, potassium voltage-gated channel subfamily E regulatory subunit 5; LQTS, long QT syndrome; LQT1, long QT syndrome 1; LQT2, long QT syndrome 2; LQT3, long QT syndrome 3; *MYH7*, myosin heavy-chain 7; *MYBPC3*, myosin binding protein C3; RCM, restrictive cardiomyopathy; *RYR2*, ryanodine receptor 2; *SCN5A*, sodium voltage-gated channel alpha subunit 5; TTN, titin; LMNA, Lamin A/C; *TNNT2*, troponin T2 cardiac type; *TNNI3*, troponin I3 cardiac type

Cardiac diseases	Molecular genetic testing of mutations in genes
LQTS	LQT1 (*KCNQ1* gene mutation), LQT2 (*KCNH2*), LQT3 (*SCN5A*) [56]; *KCNQ1*, *KCNH2*, and *SCN5A* genes, encoding the α subunit of the cardiac potassium channel Kv7.1; the α subunit of the cardiac *hERG* potassium channel and that of the cardiac sodium channel Nav1.5, respectively, involved in about 75% of LQTS patients [57]
BrS	*SCN5A* exonic coding region; with an AD pattern; *KCNE5*-related Brugada syndrome (Locus *Xq23*), which is inherited in an X-linked manner [48]
CPVT	*RYR2* gene, encoding the Ryanodine receptor 2, is involved in about 60% of patients (AD pattern); *CASQ2* gene, encoding the Calsequestrin-2, is involved in about 5% of patients (AR inheritance) [58]
HCM	*MYH7* and *MYBPC3*, encoding the sarcomeric proteins β-myosin heavy chain and cardiac myosin-binding protein C, respectively [59]
DCM	TTN, LMNA, *MYH7*, *TNNT2*, *MYBPC3*, and *SCN5A* [60]
RCM	*MYH7* and *TNNI3* genes [61]

Functional Cardiac Imaging During Exercise Stress

Exercise stress echocardiography is a relatively new approach to detecting subclinical diseases. Some of the indications include systolic and diastolic function, valve function, myocardial exercise reserve, modest wall motion anomalies, and dyssynchrony [47].

Mobile monitoring: Children can use cableless mobile and app-based gadgets in the clinic, and preliminary findings on feasibility and diagnostic values are encouraging [11]. For the pediatric athlete, mobile monitoring should be used to evaluate cardiac symptoms during exercises, such as paroxysmal symptoms of palpitations, dizziness, and syncope, as well as postintervention (e.g., arrhythmia ablation) monitoring and risk assessment (e.g., cardiomyopathies and congenital heart disease) [62].

Patients with recurrent or repeatable symptoms such as palpitation or syncope may benefit from long-term ECG monitoring with Holter monitors. A continuous loop monitor is the best way to assess athletes who have sporadic or infrequent symptoms. These monitors continually record a one to three-minute segment of surface ECG and then freeze the tape and record the previous few minutes of the event when a button on the device is pressed.

Other biochemical and hematological tests:* *Heavy resistance exercise causes platelet activation in vivo, as seen by increased platelet aggregation and β-thromboglobulin levels, which have long been known. Alterations in plasma volume and platelet count generated by exercise could explain some of these changes [63]. Resistance training also causes an increase in platelet count, plateletcrit, and mean platelet volume, as well as factor VIII and von Willebrand factor (vWF) antigen [63]. These have to be found. A lipid profile is recommended for athletes who are at risk of CAD.

A variety of acute alterations in various biochemical and hematological markers in marathon runners before, during, and after the race (before, within 4 hours, and 24 hours) and the concentrations of glucose, total protein, albumin, uric acid, calcium, phosphorus, serum urea nitrogen, creatinine, bilirubin, alkaline phosphatase, alanine aminotransferase, aspartate aminotransferase, total creatine kinase, creatine kinase-MB, myoglobin, and the anion gap all increased, consistent with exertional rhabdomyolysis and hemolysis [64].

The increase in WBC counts was mostly due to neutrophilia and monocytosis, with a decrease in circulating lymphocytes, which is consistent with an inflammatory response after tissue injury. In healthy people, acute exercise causes a brief activation of the coagulation system, which is accompanied by an increase in fibrinolytic capability. However, some people are at risk of thrombosis, and these people must be identified and monitored [65].

Screening abnormalities are defined as: 

· Exercise-related complaints (i.e., dyspnea, chest discomfort, palpitations, dizziness or fainting, syncope, and abnormal fatigue) and history of any cardiovascular condition

· A family history of SCA/SCD, as well as inherited or congenital cardiovascular conditions

· Abnormalities in the ECG [66] When implementing a program of broad and comprehensive adolescent screening and weighing the advantages and disadvantages (Table 6), keep the following points in mind [42]. 

**Table 6:** Advantages and disadvantages of screening. SCD, sudden cardiac death

Advantages	Disadvantages
Identifies individuals at risk and possibly prevent some SCDs	Recognizes abnormalities with no clinical significance that would limit sports participation unnecessarily
Assures that if the test results are normal, the risk is extremely low	The short-term and long-term psychological effects
Gives a better understanding of how sports affect heart structure, function, and SCD risk	Costs and issues with long-term insurance
	Several misleading false negatives

Coaches and athletes should be made aware of potential warning signs and symptoms (e.g., syncope, lightheadedness, perceived palpitations, or arrhythmias). Regular emergency drills, bystander cardiopulmonary resuscitation, and the use of automated external defibrillators have all been shown to improve overall and athletic population survival rates [67,68].

Management

Lifestyle Interventions

Athletes should be advised on lifestyle interventions to promote their cardiovascular health such as quitting smoking, choosing good nutrition, aiming for a healthy weight, reducing stress, limiting alcohol, and maintaining appropriate BP. Although dietary recommendations can be adapted to the needs of each athlete, there is substantial evidence that the Mediterranean or a plant-based diet low in saturated fats lowers the risk of atherosclerotic cardiovascular disease (ASCVD) [69,70].

Medical Therapy

Treatment options for older athletes with ASCVD: In patients with a 10-year ASCVD risk of intermediate or high, statin treatment is advised [74]. BP control is recommended, with a focus on lifestyle interventions for stage 1 hypertension and the addition of pharmacotherapy for stage 2 hypertension [75]. Daily low-dose aspirin may be considered for primary prevention in select individuals at higher ASCVD risk who are not at increased risk of bleeding, according to the American College of Cardiology and the American Heart Association (AHA) Prevention Guidelines (Table 7) [76].

**Table 7:** Therapies for treatment or prevention of VA. BrS, Brugada syndrome; CPVT, catecholaminergic polymorphic ventricular tachycardia; LQTS, long QT syndrome; VA, ventricular arrhythmia

Disease	Medical therapy
VA	Beta-blockers (e.g., metoprolol succinate and carvedilol) [44]
Cardiac channelopathies (e.g., LQTS and CPVT)	Beta-blockers (e.g., nadolol and propranolol) [44]
Congenital LQTS	Oral mexiletine [71]
Refractory ventricular tachycardia/cardiac arrest	Intravenous lidocaine [72]
BrS	Quinidine [44]
CPVT	Flecainide [73]

Implantable Cardioverter Defibrillator

A defibrillator is a powerful tool for terminating life-threatening VAs. A transvenous ICD, a subcutaneous implantable cardioverter defibrillator (ICD), a wearable cardioverter defibrillator, or an external defibrillator can all be used to give this therapy. These devices continually monitor the cardiac rhythm and give therapy when tachycardia satisfies preprogrammed detection rates and arrhythmia length [44].

Surgery

Patients with persistent VA and SCA survivors should be assessed for ischemic heart disease and, if necessary, revascularized. Repair or revascularization is advised in patients having an abnormal origin of a coronary artery anomaly suspected of being the cause of SCA [77-80].

A competitive athlete's diagnosis of a cardiovascular ailment typically leads to the athlete's discontinuation from sports due to worries about disease progression and exercise-induced SCD. Potential career-ending decisions are complex, involving medical, ethical, and legal issues, and can have severe psychosocial and economic consequences [81]. Giving an athlete the right to participate in the decision of whether or not to compete involves empowering them [82]. Three models of treatment decision-making are described with progressive levels of empowerment: 

1. A paternalistic model (The athlete has a passive role.)

2. Model of shared decision-making (Both the athlete and the physician participate in the decision.)

3. Informed decision-making (The decision is made by the athlete, while the role of the physician is solely to provide information.)

Empowering athletes may have a variety of benefits, including respect for people's rights and an individual approach to treatment. Finally, this should help to prevent the denial of life-threatening heart diseases by improving contact between athletes and specialized healthcare facilities, potentially increasing the availability of care and survival prospects if a catastrophic incident occurs during competition [82].

PPS has the support of international and national sporting organizations. However, there is a significant limitation in the need for highly trained medical personnel who have experience assessing athletes, analyzing their clinical data, and directing relevant further investigations when necessary without interfering with training regimens and sporting events. More research in these areas, including clinical, genetic, and public health perspectives, is required.

## Conclusions

In an athlete's career, primary prevention of SCD is a vital lifesaving procedure. The most common medical cause of sudden death in athletes is SCA. The most common cause of SCD among master athletes is CAD and genetic disorders in young athletes. Before the screening, it is necessary to understand the causes of SCD in athletes. It is the professional's responsibility to comprehend the concerns, inform the athletes who are at risk, and safeguard them. PPS was created to ensure an athlete’s health and safety, including the early detection of those at risk for SCD. Athletes who are at a high risk of sudden death can be predicted and treated with screening, which usually involves removing them from competitive sports and, in some cases, applying therapeutic preventive measures. Regular and long-term strenuous activities are associated with physiological cardiac modifications and discrete electrical manifestations, so experts must be able to distinguish pathological from physiological heart changes. False-positive results can lead to unnecessary extra testing and, unfortunately, the restriction of athletes who are not at risk.

To be effective, the prediction must be sensitive and cost-efficient, allowing for the detection of the most frequent and treatable medical conditions that cause sudden death and lead to a complication-reducing intervention. In addition to ECG and ultrasound investigations, others, such as hematological and biochemical tests, are required before authorization for participation in sports. Good history, physical examination, ECG, and CMR are the most accurate screening tools available; when used together, they may identify almost every person with a relevant structural or electrical abnormality that is associated with sudden death at the time of testing. Inherited heart disorders in young athletes can be detected with the help of family history and genetic studies. Interventions include lifestyle modifications and therapies for the treatment or prevention of VAs with beta-blockers, antiarrhythmics, and statins for intermediate or high-risk ASCVD. ICD is a powerful tool for ending life-threatening VA and revascularization surgeries for ischemic heart disease and coronary artery abnormalities. Coaches and athletes should be made aware of potential warning signs and symptoms. In addition to promoting athlete empowerment, there is a need for an intervention that encourages athletes with cardiovascular problems to compete without interfering with their training schedules or sporting events.
